# Role of cytoskeleton-related proteins in the acrosome reaction of *Eriocheir sinensis* spermatozoa

**DOI:** 10.1186/s12863-023-01112-x

**Published:** 2023-02-14

**Authors:** Yulian Tang, Lishuang Sun, Shu Li, Huiting Liu, Lvjing Luo, Zhengyu Chen, Genliang Li

**Affiliations:** Youjiang Medical University for Nationalities, Baise, 533000 Guangxi China

**Keywords:** *Eriocheir sinensis*, Spermatozoa, Acrosome reaction, Cytoskeleton-related protein, miRNA

## Abstract

Cytoskeleton-related proteins are essential for cell shape maintenance and cytoskeleton remodeling. The spermatozoa of *Eriocheir sinensis* (Chinese mitten crab) have a unique cellular structure, and the mechanism of spermatozoal metamorphosis during the acrosome reaction is not well understood. In this study, the *E. sinensis* spermatozoa were induced using calcium ionophore A23187 to undergo the acrosome reaction in vitro, and the acrosome-reacting and fresh (non-reacting) spermatozoa were collected separately. The differential expression of cytoskeleton-related protein genes in acrosome-reacting and fresh spermatozoa of *E. sinensis* was analyzed by whole transcriptome sequencing and bioinformatics analysis, and PPI network and miRNA-mRNA regulation network were constructed to analyze their possible function and regulation mechanism. The results showed that numerous differentially expressed cytoskeleton-related protein genes, miRNAs and lncRNAs were found in acrosome-reacting and fresh spermatozoa of *E. sinensis*; 27 cytoskeleton-related protein genes were down regulated and 687 miRNAs were up regulated in acrosome-reacting spermatozoa; 147 miRNAs target these 27 cytoskeleton-related protein genes. In the PPI networks, RAC1, BCAR1, RDX, NCKAP1, EPS8, CDC42BPA, LIMK1, ELMO2, GNAI1 and OCRL were identified as hub proteins. These proteins are mainly involved in the regulation of cytoskeleton organization, actin cytoskeleton organization, microtubule skeleton organization and small GTPase-mediated signal transduction and other biological processes, and play roles in pathways such as actin cytoskeletal regulation and axon guidance. miR-9, miR-31 and two novel miRNAs in the miRNA-mRNA regulatory network are the core miRNAs targeting cytoskeleton-related protein genes. miR-9 targets and regulates *OBSCN*, *CDC42BPA*, *ELMO2*, *BCAS3*, *TPR* and *OCRL*; while miR-31 targets and regulates *CDC42BPA* and *TPR*. This study provides a theoretical basis for revealing the mechanism of acrosome reaction under the special spermatozoa morphology of *E. sinensis*.

Acrosome reaction is a necessary event prior to sperm-egg fusion and is essential for fertilization to occur [1]. The spermatozoa of *Eriocheir sinensis* (Chinese mitten crab) are distinct from those of other mammals, with a unique cellular structure that is characterized by the absence of flagella, an extra-large acrosome capsule and a "cup-shaped" spermatozoa nucleus [2, 3]. Therefore, the mechanism of the acrosome reaction has attracted much attention from researchers. Cytoskeleton consists of actin filaments, microtubules and intermediate filaments, which play a key role in cell morphology maintenance and cell motility [4]. Cytoskeleton-related proteins, as a class of proteins that regulate the dynamic stability of the cytoskeleton, include tubulin, actin, myosin, and many others structural unit accessory proteins. Currently, research on cytoskeleton-related proteins has focused on humans, mice, guinea pigs, bovines and other mammals [5–9]. Cytoskeleton-related proteins are involved in regulating cytoskeleton remodeling, determining and controlling cell morphology, and also in the transport of intracytoplasmic components [10–12]. In mammals, cytoskeletal remodeling is thought to be necessary for mammalian sperm capacitation and acrosome reaction [13, 14]. Similar to mammalian spermatozoa, *E. sinensis* spermatozoa also undergo dramatic morphological changes and cytoskeleton remodeling during the acrosome reaction. The acrosome reaction of *E. sinensis* spermatozoa include radial arm contraction, acrosome vesicle outgrowth, acrosome tubule protrusion and lamellar structure shedding [15]. In this series of changes, cytoskeleton-related proteins may play important roles. However, since the role of cytoskeleton-related proteins in the acrosome reaction of *E. sinensis* spermatozoa is less reported, it is not clear which cytoskeleton-related proteins are involved in the acrosome reaction of *E. sinensis* spermatozoa, nor do we know the processes involved and the manner in which they are regulated. Therefore, the exploration of expression changes in cytoskeleton-related proteins and their regulatory mechanisms during the acrosome reaction of *E. sinensis* spermatozoa can help to reveal the mechanism of spermatozoal metamorphosis during the acrosome reaction in the special spermatozoa morphology of *E. sinensis*.

Accordingly, we induced acrosome reaction of *E. sinensis* spermatozoa in vitro and collected acrosome-reacting spermatozoa and fresh (non-reacting) spermatozoa separately. Then, Whole transcriptome sequencing and bioinformatics analysis were used to analyze the differential expression of cytoskeleton-related protein genes in acrosome-reacting spermatozoa and fresh spermatozoa. We also constructed protein–protein interaction (PPI) network and miRNA-mRNA regulatory network to analyze their possible functional roles and regulatory mechanisms. This study can provide valuable information for the reproduction research of *E. sinensis* and provide a certain theoretical basis for animal developmental biology research in the future.

## Materials and methods

### Experimental animals

Chinese mitten crabs were purchased from Ice King Aquatic Products Sales Co., Ltd. in Suqian City, Jiangsu Province, China ([SYXK (Su) 2018–0021]). A total of 30 male crabs with a body weight of 100-150 g, healthy limbs and active movement were selected and randomly divided into 2 groups. There were 18 in the experimental group (group JA) and 12 in the control group (group JO). Then they were fully aerated under tap water for 1 day. The water temperature was 24℃, pH was 6.8, and dissolved oxygen > 5.0 mg/L. The isolation and extraction of vas deferens tissues were performed at the Experimental Animal Center of Youjiang Medical University for Nationalities ([SYXK(GUI)2017–0004]).

### Main reagents and instruments

Total RNA extraction kit (Beijing Solarbio Science & Technology Co., Ltd.), Ultramicro ultraviolet spectrophotometer (Shanghai Puyuan Instrument Co., Ltd.), single-double ultra-clean workbench (Jiangsu Sujing Group Co., Ltd.), biological microscope (Shanghai Leica Instruments Co., Ltd.), desktop high-speed refrigerated centrifuge (Eppendorf China Co., Ltd.), 15 ml glass tissue grinder (Guangxi Yanpu Biotechnology Co., Ltd.).

### Acrosome-reacting spermatozoa and fresh spermatozoa preparation

A small amount (about 100 mg) of vas deferens tissue was taken from each Chinese mitten crab and quickly washed several times with calcium-free artificial seawater buffer of pH 8.1. The cleaned vas deferens tissues were mixed into one sample of every 6 animals in the experimental group and one sample of every 4 animals in the control group. Then the spermatozoa pods were gently squeezed out with forceps to prepare a spermatozoa pod suspension, and the supernatant was discarded after standing for 10 min; the spermatozoa pods precipitate was added to a 15 ml clean centrifuge tube and quickly resuspended with calcium-free artificial seawater buffer; then transferred to a glass tissue homogenizer to gently grind the spermatozoa pods about 40 ~ 50 times in order to induce the release of spermatozoa in the pods. Then, spermatozoa pod tissue fragments were removed after centrifugation at 500 r/min for 10 min, and spermatozoa were collected after centrifugation at 4000 r/min for 10 min. In the spermatozoa precipitate of the control group, 10 times the volume of RNA preservation solution was added and mixed, and permeated through 2 h of room temperature to prepare the fresh spermatozoa. In the spermatozoa precipitate of the experimental group, an appropriate amount of calcium ionophore A23187 was added, and then frozen overnight in a -80 °C refrigerator, and the acrosome reaction was observed the next day. After the acrosome reaction was successful, centrifuged at 4000 r/min for 10 min to obtain the precipitate of the acrosome-reacting spermatozoa. Then, 10 times the volume of RNA preservation solution was added to the precipitate of the acrosome-reacting spermatozoa, mixed well and infiltrated at room temperature for 2 h to serve as the acrosome-reacting spermatozoa for subsequent experiments.

### RNA extraction, sequencing and data analysis

Take the prepared suspension of acrosome-reacting spermatozoa and fresh spermatozoa, three samples in each group, centrifuge at 4000 r/min for 10 min to discard the RNA preservation solution. Total RNA was extracted using a total RNA extraction kit and sent to Shenzhen Huada Gene Technology Co., Ltd. for whole transcriptome sequencing. Sequencing quality assurance and depth was performed as usual. The preliminary analysis of the raw sequencing data was done by Shenzhen Huada Gene Technology Co., Ltd.

### Analysis of differentially expressed genes

Differentially expressed genes (DEGs), miRNAs and lncRNAs in acrosome-reacting spermatozoa and fresh spermatozoa from the sequencing data were analyzed. Differential folds were selected as Log2FC(JO/JA) ≥ 1 or ≤ -1, and *P* < 0.05 as the screening criteria for differential genes. The official gene names (Official Symbol) were annotated by comparison with the Chinese mitten crab reference genome, and the DEGs that were down regulated in acrosome-reacting spermatozoa were screened.

### Screening of cytoskeleton-related proteins

The Gene Ontology and GO Annotations database (https://www.ebi.ac.uk/QuickGO/) was searched for "sperm cytoskeleton proteins". Cytoskeleton-related GO terms of mouse, nematode, drosophilid fly and other model organisms as well as human were selected, and the cytoskeleton-related protein genes in each GO term were downloaded and extracted. All genes were summarized after removing duplicate genes. Further compared with the differential genes that are down-regulated expressed in the acrosome-reacting spermatozoa from our sequencing data, we finally screened the cytoskeleton-related protein genes in acrosome-reacting spermatozoa and fresh spermatozoa of *E. sinensis.*

### Functional enrichment of cytoskeleton-related protein genes and PPI network construction

The GO Annotations of candidate genes were obtained by mapping candidate genes to background gene sets using the R package org.HS.eg.db (version 3.1.0), and enrichment analysis was performed using the R package ClusterProfiler (version 3.14.3) to obtain enrichment analysis results, setting the minimum gene set number to 5, the maximum gene set number to 5000, *P* < 0.05, and FDR < 0.01. The protein–protein interaction (PPI) network was constructed using the STRING database (https://cn.string-db.org/), setting the minimum confidence limit equal to 0.15 and selecting the default for other parameters.

### miRNA screening and miRNA-mRNA regulatory network construction for targeting cytoskeleton-related protein genes

TargetScan (http://www.targetscan.org/vert_71/), miRanda (http://www.microrna.org/microrna/home.do/) and RNAhybrid (http://bibiserv.techfak.uni-bielefeld.de/rnahybrid/) were used to predict and analyze the miRNAs regulating the above cytoskeleton-related protein genes. The miRNA-mRNA regulatory network was constructed and visualized using Cytoscape software, and the hub genes in the network were analyzed using the cytoHubba plugin, by assigning and ordering each gene with the topological network algorithm.

### Statistical analysis

The data of this study were analyzed using SPSS 23.0 statistical software. Continuous normal distribution of measurement data was statistically described by mean ± standard deviation. Independent samples t-test was used for analysis, and differences were considered statistically significant at *P* < 0.05.

## Results

### Acrosome-reacting spermatozoa and fresh spermatozoa

**Fig. 1:**
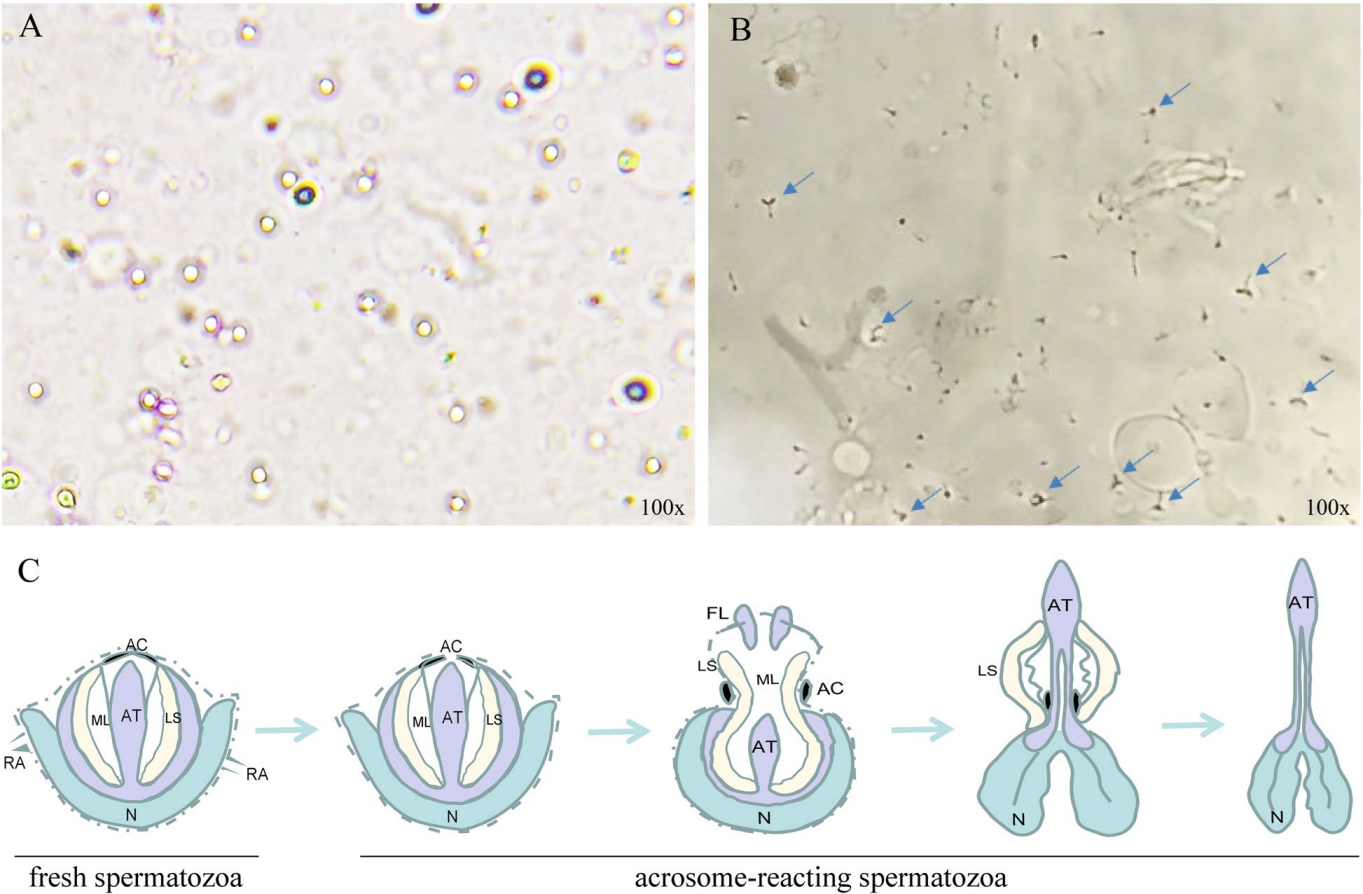
Acrosome-reacting spermatozoa and fresh spermatozoa in *Eriocheir sinensis*. **A** fresh spermatozoa; **B** acrosome-reacting spermatozoa; **C** Diagram for the process of acrosome reaction in spermatozoa. The process of acrosome reaction can be divided into four stages: (1) the contraction of radial arm and the breaking to form an aperture on apical cap; (2) the reversion of acrosome vesicle; (3) the extension of acrosome tubule; (4) the shrinking and disappearance of lamellar structures. RA: Radial arm; AC: Apical cap; AT: Acrosome tubule; LS: Lamellar structures; ML: Middle layer; FL: Fribrous layer; N: Nucleus

The spermatozoa of *E. sinensis* were induced to undergo acrosome reaction in vitro, and the results are shown below (Fig. 1). When acrosome reaction occurs in spermatozoa, the radial arm contracts, the apical cap breaks to form an aperture, the acrosome vesicle reverses, the acrosome tubule extends forward, and then the lamellar structure shrinks and disappears, finally forms the structures as shown in the figure (Fig. 1B), such as a Y-shaped fork; but fresh (non-reacting) spermatozoa are round (Fig. 1A). The diagram for the process of acrosome reaction occurring in the spermatozoa of *E. sinensis* was shown in Fig. 1C.

### Analysis of differentially expressed genes between acrosome-reacting spermatozoa and fresh spermatozoa

**Fig. 2:**
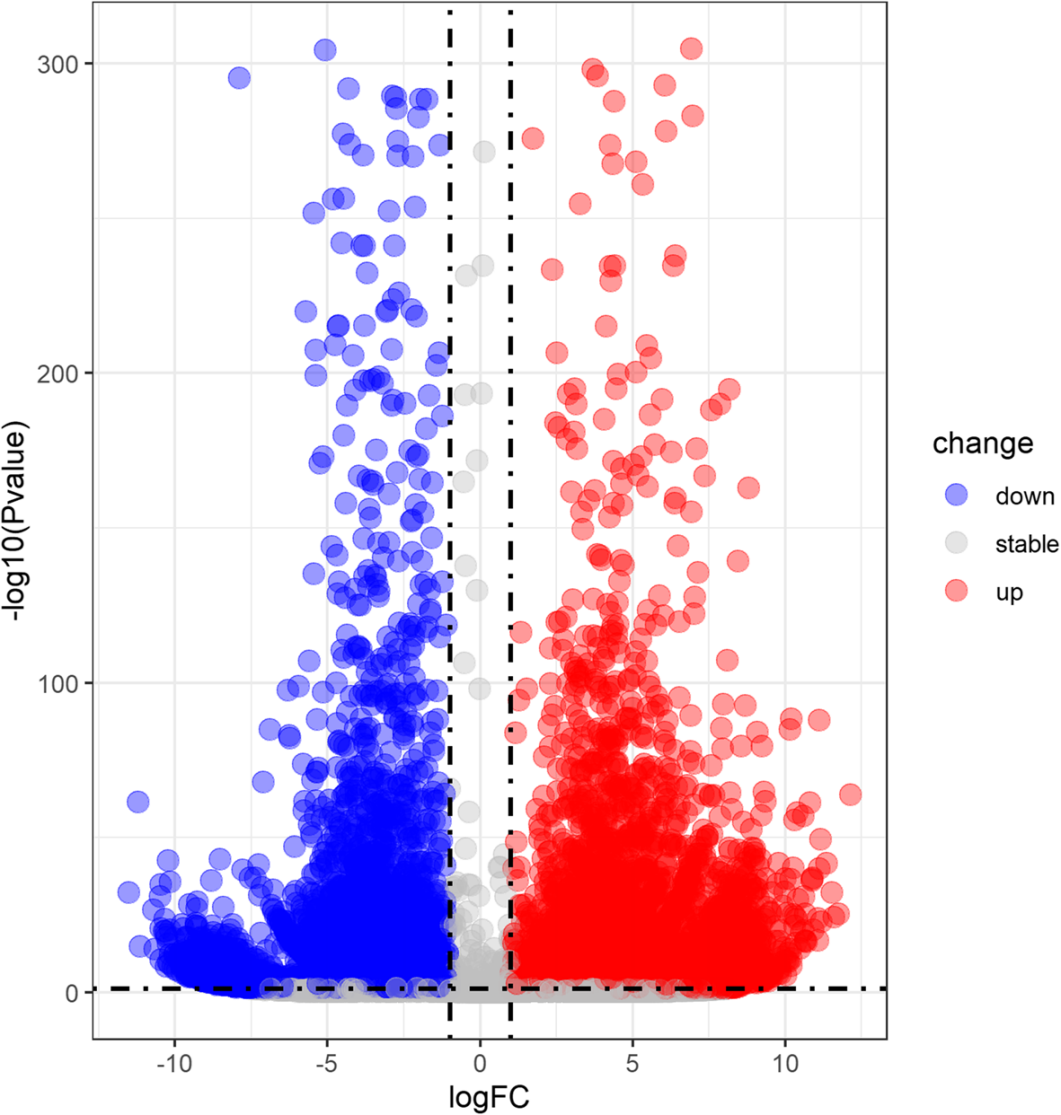
Volcano Plot of differentially expressed genes (DEGs) in the sample of acrosome-reacting spermatozoa and fresh spermatozoa of *Eriocheir sinensis*. Up-regulated genes are in red, down-regulated genes are in blue, non-differentially expressed genes are in gray

Through whole transcriptome sequencing of acrosome-reacting spermatozoa and fresh spermatozoa, a total of 6510 up-regulated genes, 8028 down-regulated genes and 9942 non-differentially expressed genes were identified in the two samples. The Volcano Plot of DEGs shows these genes (Fig. 2). Among them, 849 mRNAs were down regulated in acrosome-reacting spermatozoa and up regulated in fresh spermatozoa; on the contrary, 687 miRNAs were up regulated in acrosome-reacting spermatozoa and down regulated in fresh spermatozoa.

### Screening of cytoskeleton-related protein genes

**Fig. 3:**
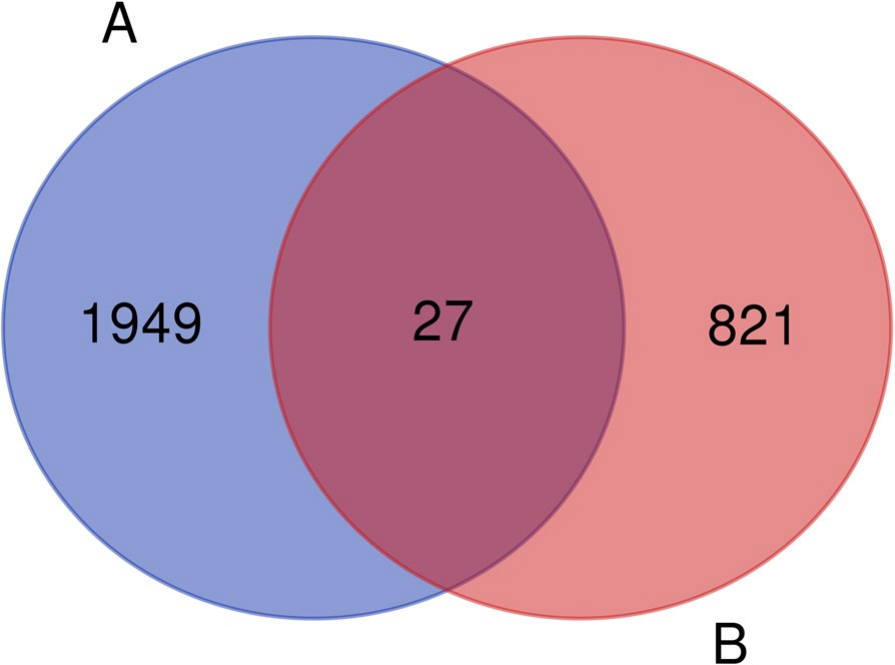
Intersection of cytoskeleton-related protein genes in the database and differentially expressed genes in whole transcriptome sequencing data. Gene set A comes from Gene Ontology and GO Annotation database, with a total of 1976 genes; Gene set B comes from whole transcriptome sequencing of acrosome-reacting spermatozoa and fresh spermatozoa of *Eriocheir sinensis*, with a total of 849 genes

After the search of Gene Ontology and GO Annotation database, a total of 10 GO terms directly related to the spermatozoa cytoskeleton were retrieved, and their annotated gene numbers are shown in Table 1. 1976 genes were obtained after removing duplicate genes. Further comparison with 849 genes that were down regulated in acrosome-reacting spermatozoa from the sequencing data, finally yielded 27 cytoskeleton-related protein genes (Fig. 3). They were *EPS8*, *OCRL*, *DCP2*, *SSH1*, *CHMP5*, *CUL3*, *LIMK1*, *TAOK1*, *CORO6*, *NCKAP1*, *CYLD*, *CALD1*, *BCR*, *GNAI1*, *TPR*, *FKBP4*, *BCAS3*, *RAC1*, *RAE1*, *KAT2A*, *ELMO2*, *ALDOA BCAR1*, *CDC42BPA*, *OBSCN*, *RDX and CALR.*

**Table 1:** Number of genes annotated by the 10 GO terms related to cytoskeleton in Gene Ontology and GO Annotation database

Number	GO Terms	Functional annotation	Number of genes
1	GO:0051493	regulation of cytoskeleton organization	1071
2	GO:0051495	positive regulation of cytoskeleton organization	319
3	GO:0051494	negative regulation of cytoskeleton organization	318
4	GO:0030036	actin cytoskeleton organization	1294
5	GO:0031532	actin cytoskeleton reorganization	128
6	GO:0032956	regulation of actin cytoskeleton organization	715
7	GO:2000251	positive regulation of actin cytoskeleton reorganization	26
8	GO:2000250	negative regulation of actin cytoskeleton reorganization	1
9	GO:2000249	regulation of actin cytoskeleton reorganization	53
10	GO:1903119	protein localization to actin cytoskeleton	4

### Functional enrichment and PPI network of cytoskeleton-related proteins

**Fig. 4:**
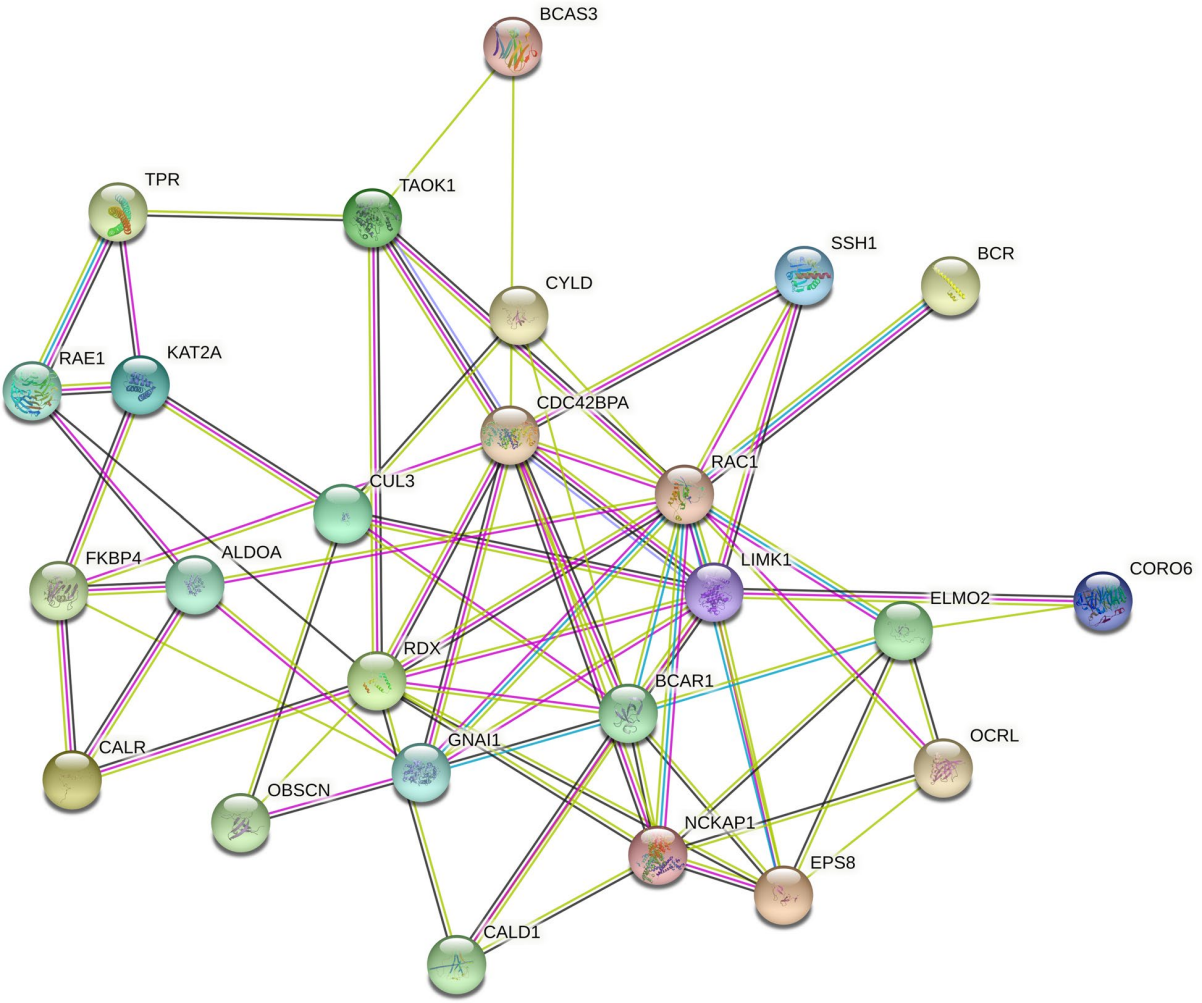
The protein–protein interaction (PPI) network of 27 cytoskeleton-related proteins

**Fig. 5:**
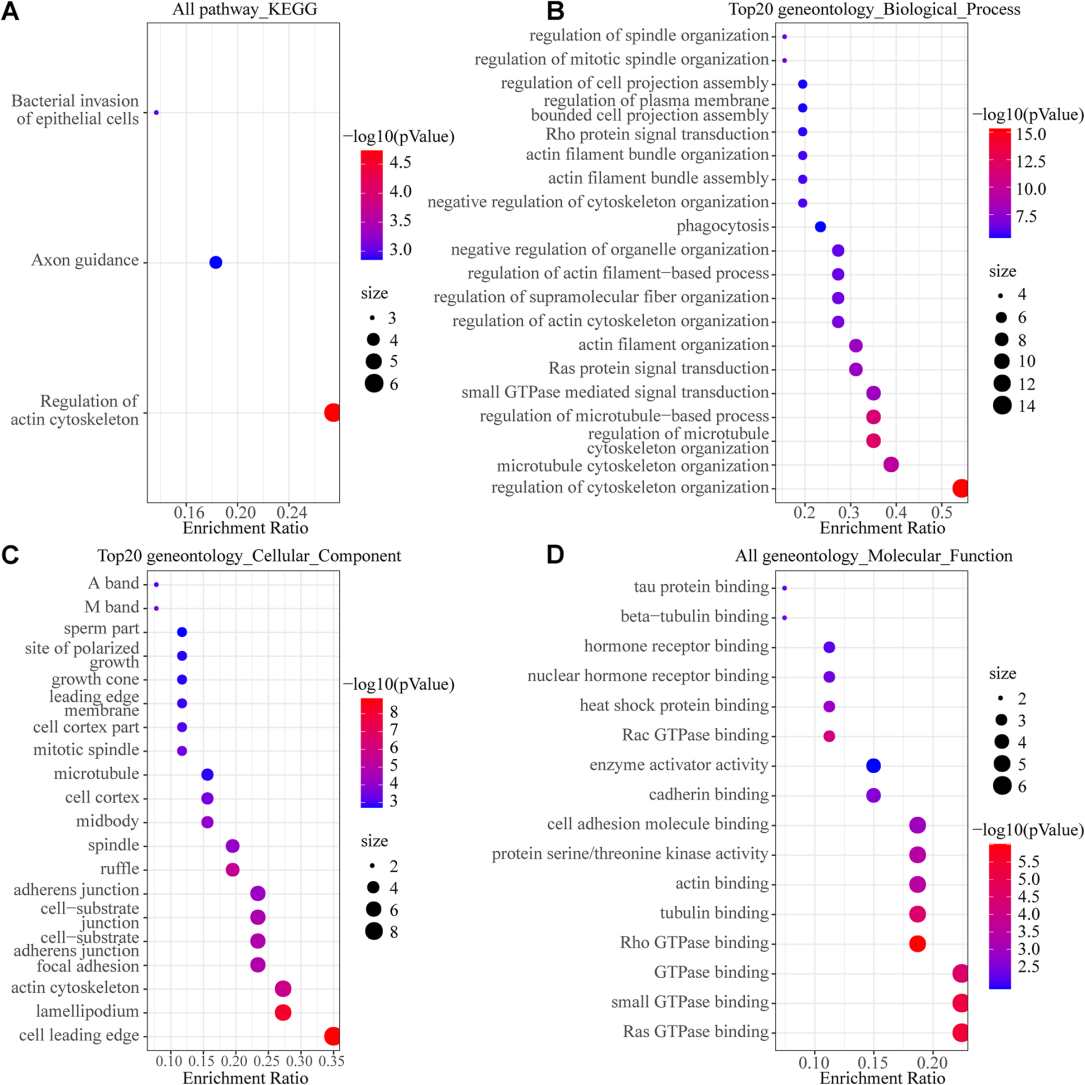
GO and KEGG enrichment analysis of 27 cytoskeleton-related protein genes. **A** all KEGG pathways in which the 27 cytoskeleton-related protein genes are involved [16]; **B** Top20 biological processes in which the 27 cytoskeleton-related protein genes are involved; **C** Top20 cellular components in which the 27 cytoskeleton-related protein genes are located; **D** all molecular functions of the 27 cytoskeleton-related protein genes

The PPI network was constructed for 27 cytoskeleton-related proteins by STRING database, and the results showed that they had strong interactions (Fig. 4). Among them, 10 proteins, including RAC1, BCAR1, RDX, NCKAP1, EPS8, CDC42BPA, LIMK1, ELMO2, GNAI1 and OCRL, were the hub proteins in the network. Functional enrichment analysis of the 27 cytoskeleton-related protein genes using the R package showed that they mainly have molecular functions such as Ras GTPase binding, small GTPase binding, GTPase binding, Rho GTPase binding, tubulin binding, actin binding, protein serine/threonine kinase activity and cell adhesion molecule binding, etc.; mainly in the cell leading edge, lamellipodium, actin cytoskeleton, focal adhesion, spindle, microtubules, site of polarized growth, A band and M band of spermatozoa, etc.; mainly involved in the regulation of cytoskeleton organization, microtubule cytoskeleton organization, regulation of actin cytoskeleton organization, small GTPase mediated signal transduction, Ras protein signal transduction, regulation of plasma membrane bounded cell projection assembly, regulation of cell projection assembly, cell protrusion organization, regulation of mitotic spindle organization and other biological processes; the pathways involved are mainly enriched in actin cytoskeleton regulation and axon guidance (Fig. 5).

### miRNAs targeting cytoskeleton-related protein genes and miRNA-mRNA regulatory network

**Fig. 6:**
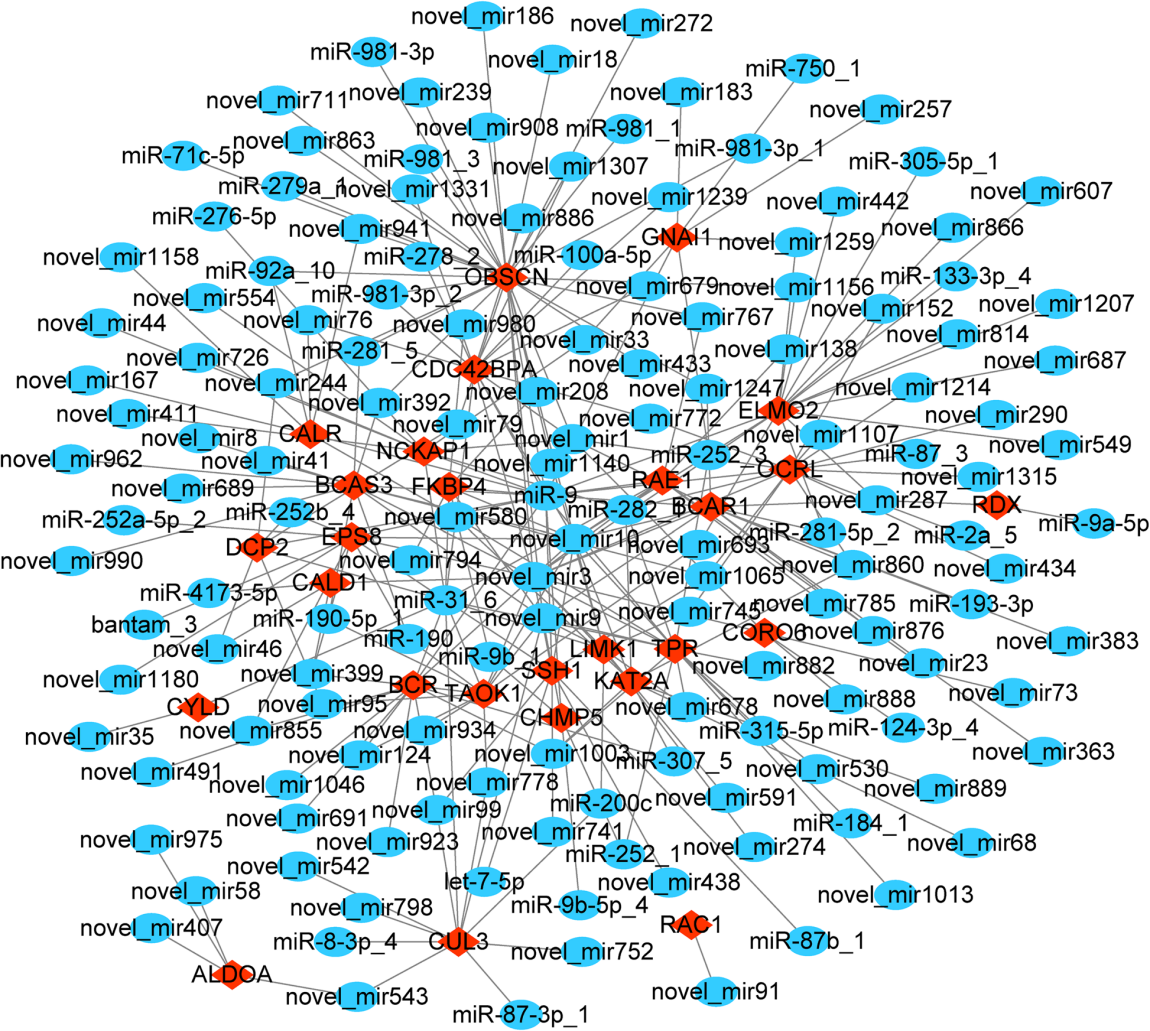
miRNA-mRNA regulatory network consisting of 27 cytoskeleton-related protein genes and 147 miRNAs. mRNA is indicated by red diamonds, miRNA is indicated by blue ovals; the miRNAs beginning with “novel” have not yet been named in *Eriocheir sinensis*

**Fig. 7:**
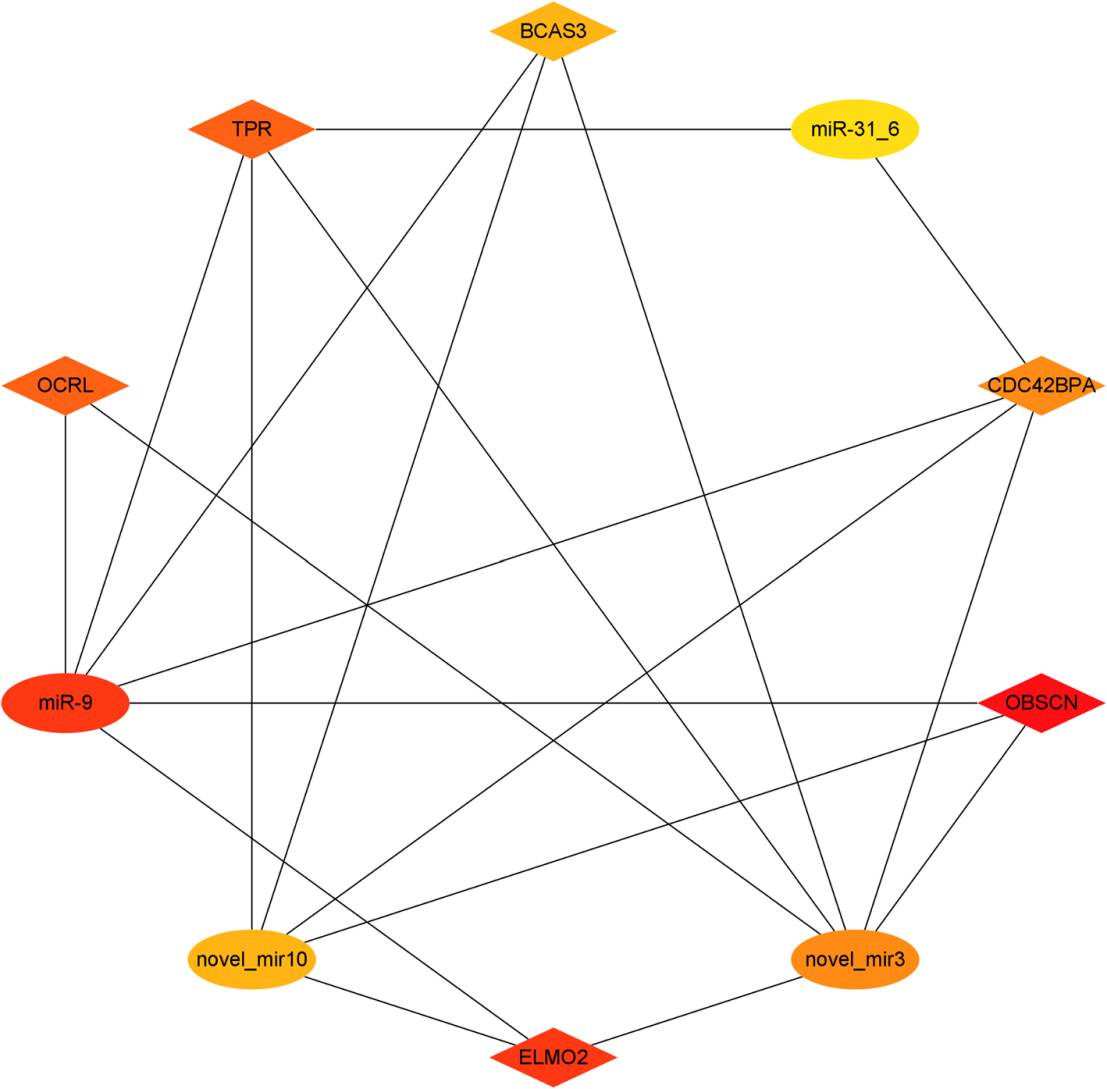
Interactions between 4 core miRNAs and 6 core cytoskeleton-related protein genes. mRNA is indicated by diamonds, miRNA is indicated by ovals; the miRNAs beginning with “novel” are unnamed miRNAs in *Eriocheir sinensis*

Based on the three databases of TargetScan, miRanda and RNAhybrid, the miRNAs targeting cytoskeleton-related protein genes were comprehensively predicted. It was finally found that 147 miRNAs and the 27 cytoskeleton-related protein genes may have targeted regulatory relationships. They formed a total of 276 targeted regulatory relationship pairs, which formed a strong miRNA-mRNA regulatory network (Fig. 6). Among them, 4 miRNAs (miR-9, miR-31, novel_mir10 and novel_mir3) and 6 genes (*OBSCN*, *CDC42BPA*, *ELMO2*, *BCAS3*, *TPR* and *OCRL*) constituted the core of the network, and they interacted with each other (Fig. 7). The core miRNAs in the network, miR-9, miR-31, novel_mir10 and novel_mir3, all have target-directed actions on multiple cytoskeleton-related protein genes. miR-9 has a possible target regulation relationship with *OBSCN*, *CDC42BPA*, *ELMO2*, *BCAS3*, *TPR* and *OCRL*; miR-31 has a possible target regulation relationship with *CDC42BPA* and *TPR*. In addition, the miRNAs targeting the top10 core genes in the PPI network in this miRNA-mRNA regulatory network are shown in Table 2.

**Table 2:** The miRNAs targeting top10 genes in PPI network

number	mRNA	Target miRNA
1	*RAC1*	novel_mir91
2	*BCAR1*	novel_mir9、novel_mir876、novel_mir860、novel_mir383、novel_mir3、novel_mir287、novel_mir23、novel_mir1247、novel_mir10、novel_mir1、miR-9、miR-281-5p_2、miR-193-3p、
3	*RDX*	novel_mir287、miR-9a-5p
4	*NCKAP1*	novel_mir726、novel_mir554、novel_mir44、novel_mir411、novel_mir33、novel_mir1、miR-9、miR-31_6、miR-282_1、miR-276-5p
5	*EPS8*	novel_mir855、novel_mir399、novel_mir3、novel_mir1180、novel_mir10、miR-9、miR-31_6、miR-31_6、miR-31_6、miR-252b_4、miR-252a-5p_2、miR-190、bantam_3
6	*CDC42BPA*	novel_mir886、novel_mir772、novel_mir76、novel_mir679、novel_mir3、novel_mir1331、novel_mir1307、novel_mir1239、novel_mir10、novel_mir1、miR-981-3p_2、miR-9、miR-31_6、miR-31_6、miR-278_2、miR-100a-5p
7	*LIMK1*	novel_mir9、novel_mir745、novel_mir678、novel_mir591、novel_mir3、novel_mir274、novel_mir1140、novel_mir10、miR-31_6、miR-200c
8	*ELMO2*	novel_mir866、novel_mir814、novel_mir693、novel_mir687、novel_mir607、novel_mir549、novel_mir442、novel_mir3、novel_mir287、novel_mir1259、novel_mir1207、novel_mir1156、novel_mir10、miR-9、miR-305-5p_1、miR-281-5p_2、miR-133-3p_4
9	*GNAI1*	novel_mir33、novel_mir257、novel_mir183、novel_mir1259、miR-9、miR-750_1、miR-252_3
10	*OCRL*	novel_mir860、novel_mir772、novel_mir745、novel_mir434、novel_mir3、novel_mir290、novel_mir152、novel_mir1315、novel_mir1214、novel_mir1107、novel_mir1065、novel_mir1、miR-9、miR-87_3、miR-2a_5、miR-282_1

The miRNAs beginning with “novel” in the table are unnamed miRNAs in *Eriocheir sinensis*

## Discussion

The unique cytoskeleton can not only regulate cell shape, structure, stability, plasticity and dynamic response to environmental stimuli, but also maintain the biological function of certain organs [17]. The spermatozoa of *E. sinensis* have a cytoskeleton structure that is significantly different from that of normal mammalian spermatozoa. The spermatozoa are nearly spherical, tailless, with uneven surface, numerous radiating arms, and a huge spherical acrosome, which compresses the nucleus into a cup shape [18, 19]. This special cytoskeletal structure determines that the process of the acrosome reaction is also different: contraction of the radial arms, outgrowth of the acrosome capsule, forward extension of the acrosome canal, and finally shedding of the lamellar structure [15]. Each of these steps is inseparable from cytoskeleton remodeling, and its spermatozoa cytoskeleton-related proteins should play a significant role. Using the cytoskeleton-related proteins of model organisms such as mouse, nematode, drosophilid fly and other model organisms as well as human as the background, we screened out cytoskeleton-related protein genes from the acrosome-reacting spermatozoa and fresh spermatozoa of *E. sinensis*. And we further analyzed their differential expression and function in the acrosome-reacting spermatozoa and fresh spermatozoa of *E. sinensis*, and the miRNAs targeting them. The study will help to better understand their possible regulatory mechanisms in the acrosome reaction of *E. sinensis* spermatozoa.

Through whole transcriptome sequencing, we found a very large number of differentially expressed genes between acrosome-reacting spermatozoa and fresh spermatozoa of *E. sinensis*, and many cytoskeleton-related protein genes were up regulated in fresh spermatozoa and down regulated in the acrosome reaction of *E. sinensis* spermatozoa. The reasons for the down-regulated expression of cytoskeleton-related protein genes in *E. sinensis* spermatozoa are poorly understood. microRNAs (miRNAs) are an important class of endogenous factors with the ability to regulate the expression of protein-coding genes. In recent years, it has been found that its role in testicular development and spermatogenesis has become more and more extensive [20, 21]. They involved in various biological processes such as male germ cell differentiation, gonadal oxidative stress, and testicular immune homeostasis through targeted inhibition of gene expression [22, 23], and can be considered as crucial regulators of biological processes [24]. We found numerous miRNAs differentially expressed between acrosome-reacting spermatozoa and fresh spermatozoa, many of which were up regulated in acrosome-reacting spermatozoa of *E. sinensis*. Through the prediction of miRNAs and the construction of miRNA-mRNA regulatory network, we found that 147 miRNAs and 27 cytoskeleton-related protein genes have possible target regulatory relationships. They formed 276 pairs of targeted regulatory relationships. Among the 276 targeted regulatory relationship pairs formed, miR-9 and *OBSCN*, *CDC42BPA*, *ELMO2*, *BCAS3*, *TPR*, and *OCRL*, and miR-31 and *CDC42BPA* and *TPR* constituted the core of the targeted regulatory relationship network. miR-9 has been reported to promote the proliferation, invasion and metastasis of various cancer cells [25–27] and also has a role in regulating spermatogenesis of spermatogonia [25]. It is evident that miR-9 has a significant pro-differentiation effect on cells, but its involvement in acrosome reaction has not been reported. miR-31 has been reported to regulate apoptosis and invasion of hepatocellular carcinoma HepG2 cells through the ROCK1/F-actin pathway [28]. It can be seen that miR-31 can regulate cytoskeleton-related protein genes involved in the physiological functions of cells, but its study in acrosome reaction has also not been mentioned yet. Acrosome reaction of spermatozoa is a special physiological process, especially the acrosome reaction of *E. sinensis* spermatozoa has a different reaction process from that of general mammals. In the present study, the target gene of miR-9, Obscurin, cytoskeletal calmodulin and titin-interacting RhoGEF (*OBSCN*), a cytoskeletal calmodulin, can mediate direct interactions between the sarcoplasmic reticulum and sarcoplasmic fibers by participating in the organization of sarcoplasmic fibers during cytoskeletal assembly [29]; CDC42 binding protein kinase alpha (CDC42BPA), which can mediate Cdc42-MRCK signaling to induce peripheral actin formation and promote cytoskeleton reorganization [30]; BCAS3 microtubule associated cell migration factor (BCAS3), a microtubule associated cell migration factor, has been reported to promote cytoskeletal reorganization by coupling microtubules and intermediate filaments [31]. However, whether the specific roles of these protein genes in the unique acrosome reaction of *E. sinensis* spermatozoa are different? We don't know yet. Therefore, how miR-9 and miR-31 targeting these cytoskeleton-related protein genes are involved in the acrosome reaction of *E. sinensis* spermatozoa deserve subsequent in-depth study.

We know that the biological functions of miRNAs mainly act through their target genes. miRNAs target genes, and these target genes perform specific functions due to their own special domains, thereby participating in biological processes. In our present study, we found that 27 cytoskeleton-related protein genes were down regulated in acrosome reaction of *E. sinensis* spermatozoa, and the proteins encoded by *RAC1*, *BCAR1*, *RDX*, *NCKAP1*, *EPS8*, *CDC42BPA*, *LIMK1*, ELMO2, *GNAI1* and *OCRL* are hub proteins in the PPI network of 27 cytoskeleton-related proteins. Their functional enrichment analysis revealed that they mainly have molecular functions such as Ras GTPase binding, small GTPase binding, GTPase binding, Rho GTPase binding, tubulin binding, actin binding, protein serine/threonine kinase activity and cell adhesion molecule binding, etc. Ras-GTPase, an activating protein, is considered an important putative of Ras inactivation and plays an important role in tissue homeostasis [32]. Local GTPase activation has been reported to lead to the exposure of effector structural domains, which upon binding to effector proteins can initiate downstream signaling [33]. G protein subunit alpha i1 (GNAI1) which has GTPase enzymatic activity can bind to GTPase activating protein, and play an important role in signal transduction [34]. Small GTPases control the signal transduction of transforming growth factor beta (TGFβ), which plays an important role in receptor endocytosis and actin cytoskeleton remodeling [35]. RAS-related C3 botulinum toxin substrate 1 (RAC1) has a small GTPase-binding protein structural domain that binds to small GTPases and is involved in actin cytoskeleton remodeling in the apical region of the acrosome during guinea pig spermatozoa capacitation. The formation of the actin cytoskeleton in the apical region of the acrosome is considered a necessary event for spermatozoa capacitation and acrosome reaction in guinea pigs and is driven by *Rac1* [36]. In this study, we also found that *RAC1* is differentially expressed in the acrosome-reacting spermatozoa of *E. sinensis*, suggesting that *RAC1* may also regulate actin cytoskeleton remodeling in *E. sinensis* spermatozoa through its own specific structural domain and participate in the process of acrosome reaction. In addition, the extension and contraction of membrane protrusions and adhesion are controlled by the Rho family of small GTPases [37]. Rho GTPase, as the structural basis of signaling, has been reported to be directly related to miRNAs and regulated by miRNAs and covalent modifications [38]. OCRL inositol polyphosphate-5-phosphatase (OCRL) has a Rho GTPase activating protein domain and can interact with Rho GTPase in the trans-Golgi network, by activating phosphatidylinositol 4, 5-bisphosphate 5-phosphatase, thereby affecting actin polymerization [39]. When the acrosome reaction of *E. sinensis* spermatozoa occurs, the acrosome capsule will be everted and the outer membrane of the acrosome will be stretched and contracted. Therefore, proteins with such domains may play an important role in acrosome reaction.

In summary, we induced acrosome reaction of *E. sinensis* spermatozoa in vitro and found a large number of differentially expressed mRNAs and miRNAs in acrosome-reacting spermatozoa and fresh spermatozoa. By comparing with model organisms such as mouse, nematode, drosophilid fly and human cytoskeleton-related proteins, we finally found 27 down-regulated cytoskeleton-related protein genes in the acrosome-reacting spermatozoa of *E. sinensis*. They are mainly involved in the regulation of cytoskeleton organization, actin cytoskeleton organization, microtubule skeleton organization and small GTPase mediated signal transduction, acting through actin cytoskeleton regulation and axon guidance pathways. RAC1, BCAR1, RDX, NCKAP1, EPS8, CDC42BPA, LIMK1, ELMO2, GNAI1 and OCRL are the hub proteins in the PPI network of 27 cytoskeleton-related proteins. In the strong miRNA-mRNA regulatory network formed, 4 miRNAs with 6 cytoskeleton-related protein genes formed the core of the regulatory network. miR-9 had possible target regulatory relationships with *OBSCN*, *CDC42BPA*, *ELMO2*, *BCAS3*, *TPR* and *OCRL*, and miR-31 with *CDC42BPA* and *TPR*. The spermatozoa of *E. sinensis* have a unique cytoskeletal structure and the mechanism of acrosome reaction is obviously different. In this study, we investigated the expression and functional roles of cytoskeleton-related protein genes in acrosome-reacting spermatozoa and predicted the miRNAs that may regulate them, which may provide a theoretical basis for further understanding the mechanism of the acrosome reaction in *E. sinensis* spermatozoa. However, further validation of the miRNAs targeting the expression of related genes is needed to better elucidate the mechanism of acrosome response in *E. sinensis* and provide more valuable information for its reproduction and developmental biology of other animals. However, the targeting of miRNAs to cytoskeleton-related protein genes still needs to be further validated in order to provide more valuable information for their reproduction and developmental biology of other animals.

## Data Availability

The datasets generated and/or analyzed during the current study are available in the Sequence Read Archive (SRA), https://www.ncbi.nlm.nih.gov/sra/?term=PRJNA807466.
